# Insurance Day by Day

**Published:** 1912-05-11

**Authors:** 


### The Hospital

The Workers' Newspaper of Administrative Medicine and Institutional Life, Administration, National Insurance and Health.

No. 1348, Vol. LII. SATURDAY, MAY 11, 1912. PRICE ONE PENNY.

### INSURANCE DAY BY DAY.

If a month ago the Insurance Act, with all its unsolved problems for future development, seemed to be in a state of suspended animation and to be quietly ignored by politicians and the press, at all events the corpse has briskly reawakened since then, and is now so lively that almost every day sees some important sign of activity.

To begin with, a party *coup* on the night of May 1 forced the Chancellor to a formal recognition of the reality of the situation created by the firmness of the medical profession. On that date Mr. Grant moved a resolution in the House of Commons to the effect that no settlement of the dispute about medical benefit could be satisfactory unless it was acceptable to the profession. The terms of this motion were practically a vote of censure upon the Chancellor, and a Government whip was issued to ensure its rejection. However, it became apparent that there was a perceptible risk of a Ministerial defeat over it; and to avoid this the Chancellor accepted the motion with as good a grace as he could muster. It may be argued with some reason that party intrigue and manœuvre are no proper weapons wherewith to endeavour to secure justice; for the doctor's case against the Act ought to be outside the sphere of party politics. But the fact has to be faced that the Chancellor and Mr. Masterman have sided with the friendly societies for obvious reasons, and that force is the only argument they will recognise. During the debate upon this motion in Parliament Mr. Lloyd George made several statements regarding the actions of the British Medical Association and other medical bodies which were, to put it mildly, unfounded and inaccurate. Some of these, we are glad to note, were promptly nailed to the counter the next day by Dr. Alfred Cox, the newly-appointed medical secretary of the Association; and others were exposed just as promptly by Mr. Lowry, author of "Can the Doctors work the Act?"

The next sensation of the week was the divulsion on May 4 of the terms of a supplementary pledge, which is being asked by the British Medical Association from all qualified practitioners over and above that which was given, to all intents and purposes, unanimously last year. Every practitioner is to be asked to place in the hands of the local secretary his resignation of all contributory contract practice appointments in so far as they extend to insured persons, and to undertake not to accept any appointment so resigned.

The object of this drastic policy is to secure that, if necessary, all forms of "club practice" shall be terminated in every part of the kingdom on a given date, probably in January of next year. To effect this the resignations could be handed in by the local secretaries at the end of June next ensuing; unless, of course, in the meantime the demands of the profession are met satisfactorily. It will be seen that the recent lessons of the universal strike have been taken to heart by the State Sickness Insurance Committee of the British Medical Association. Recalcitrant practitioners who will not sign are to be visited by their professional neighbours and subjected to a strictly "peaceful picketing." Combined with the scheme not long ago published for providing medical attendance to the provident poor outside the Insurance Act altogether, it is clear that a new spirit is animating the Association.

We referred last week to the widely credited rumour that Mr. Lloyd George and Mr. Masterman have at last perceived that the doctors are in earnest, and have made up their minds to concede them higher rates of pay in order to save the Act. If it were otherwise, this last demonstration in force would surely convince them. Nor are signs wanting that they have decided to abandon the attitude of scornful and hectoring contempt which has been the Chancellor's mood until the last few days. The Press Association on Tuesday issued a kind of semi-official bulletin to the effect that further provision will be made for the remuneration of medical practitioners under the Insurance Act. The form of this pronouncement leaves little room for doubt that it originates from the Chancellor. Its substance seems to be an offer of a rate of seven-and-six instead of six shillings provided by the Act, as against the eight-and-six demanded. Nothing is said as to the £2 a week income limit or any other part of the "six point policy"; so it appears likely that this sop is an endeavour to buy off the profession.

The concession is certainly a considerable one, and may bribe some of the weaker vessels into deserting their brethren and destroying the unanimity of the profession; probably that is the object with which it has been bruited about. But it is so far from meeting the demands of the British Medical Association—and these, it must be remembered, were put forward, not as a basis for negotiations, but as the barest acceptable *minimum*—that it is difficult to see how that body can regard it as a complete solution of the *impasse*. Still it is evident that a considerable change has come over the views of the Chancellor, who is astute enough to recognise the jeopardy in which his Act stands and exceedingly anxious to preserve it from hopeless failure. He surrendered easily enough to the voting power of the friendly societies last year, and he will probably do the same to the doctors if only they remain as determined and united as they have lately shown themselves.

